# Selected Extended Papers of the 12^th^ International Conference on Practical Applications of Computational Biology and Bioinformatics (PACBB)

**DOI:** 10.1515/jib-2019-0004

**Published:** 2019-02-15

**Authors:** Florentino Fdez-Riverola, Miguel Rocha

**Affiliations:** ESEI: Escuela Superior de Ingeniería Informática, Edificio Politécnico, University of Vigo, Ourense, Spain; Centre of Biological Engineering, School of Engineering, University of Minho, Braga, Portugal

This special issue includes extended versions of a number of papers selected from the International Conference on Practical Applications of Computational Biology and Bioinformatics (PACBB 2018) that was held in Toledo (Spain) in June 2018. This forum, already in its twelfth edition, aims to reunite and promote the collaboration among researchers developing applied Bioinformatics or Chemoinformatics research.

The papers presented at the conference cover a large range of computational algorithms and tools, focusing on artificial intelligence methods, applied to address relevant problems from topics ranging from high-throughput data integration, analysis and mining, biological networks and models, or biomedical text mining, just to name a few topics.

The five selected papers for this issue are globally focused in the topics of data integration, machine and deep learning, model reconstruction and simulation. These include recent challenges, such as the exploitation of machine and deep learning algorithms, metabolic model integration or gene selection.

In the first paper by Perscheid et al. [1], the authors compare the performance of traditional and integrative gene selection approaches in the context of RNAseq gene expression data analysis. They propose a framework for the automatic integration of external knowledge within existing gene selection algorithms.

The second paper, by Sebastián-Pérez et al. [2] deals with the development of machine learning methods for a QSAR (quantitative structure–activity relationship) task, related with the identification of putative inhibitors of the LRRK2 protein The authors propose alternative molecular descriptors subsets and different strategies for supervised learning.

Still within the machine learning field, the third paper, by Stahl et al. [3], proposes an alternative deep convolutional neural network for the analysis of arbitrary sized graph structures representing molecules. Enabling the incorporation of global and local molecular information, showing improvements over recent alternative methods.

The paper by Vieira et al. [4] addresses the integration of genome-scale metabolic models of humans, proposing a pipeline to integrate multiple models, evaluating the presence of database identifiers and annotations for metabolites and reactions. Information was organized in a graph database allowing clustering of metabolites and reactions through their similarity.

The final paper, by Salguero et al. [5], proposes a model for tumour growth based on cellular automata, allowing load balancing of cell distribution among computational threads, which may be adjusted through specified parameters. This allows reduction in execution time and improved speedup when compared with sequential alternatives.
